# Enhancing Electrical Conductivity of Composites of Single-Walled Carbon Nanotubes and Ethyl Cellulose with Water Vapor

**DOI:** 10.3390/ma13245764

**Published:** 2020-12-17

**Authors:** Monika Rdest, Dawid Janas

**Affiliations:** 1 Department of Materials Science and Metallurgy, University of Cambridge, Cambridge CB3 0FS, UK; Monika.Rdest@gmail.com; 2 Department of Organic Chemistry, Bioorganic Chemistry and Biotechnology, Silesian University of Technology, 44-100 Gliwice, Poland

**Keywords:** carbon nanotubes, thin films, doping

## Abstract

Electrically conducting composites are highly sought-after materials. Their capacity to withstand mechanical deformation while simultaneously offering facile charge transport recently opened numerous exploitation fields for them. In this contribution, composites were made from single-walled carbon nanotubes (SWCNTs) and ethyl cellulose (EC). Then, a straightforward process of doping involving water vapor was developed and tested over 30 days. The inclusion of water in the EC/SWCNT network resulted in a notable increase in the electrical conductivity from 250 ± 21 S/cm to 905 ± 34 S/cm. Interestingly, doping of the material experienced remarkable stability due to the favorable surface chemistry of the EC filler.

## 1. Introduction

Over the last hundred years, the global consumption of primary energy increased by an order of magnitude from 17,963 TWh to 158,839 TWh annually [1], a considerable share of which is converted to electrical energy. Unfortunately, the process of generation and utilization of electrical energy is inefficient, resulting in the wasting of ca. 66% of it as waste heat [2]. Since the energy demand is envisioned to keep rising at a staggering rate, there is a need to develop materials that can improve the management of the resources that we currently have at our disposal. Increasing the efficiency of processes around us is unquestionably more ecologically friendly, rather than generating even more electrical energy from primary resources to sustain the progress of civilization.

To accomplish this challenge, we need solutions capable of overcoming the limitations of the classical materials surrounding us in our daily life. The birth of nanomaterials in the second half of the XX century marked with the famous “There is plenty of room at the bottom” speech by Richard Feynman [3] opened new perspectives to bring this aim closer to realization. The synthesis of materials with atomistic precision confirmed that they can offer new functionalities or unprecedented performance levels [4,5,6]. The invention of carbon nanostructures such as carbon nanotubes (CNTs) [7] or graphene [8] made a particularly strong impact on this front.

Individual CNTs demonstrated excellent electrical [9,10], thermal [11,12], optical [13,14], and mechanical properties [15,16], which gave hope that many technological problems will become avoidable upon their application in real life. For instance, the ability of metallic single-walled CNTs to transport charge at more than three orders of magnitude higher current density than Cu, reaching the values of 4 × 10^9^ A/cm^2^, demonstrated that they can be considered as viable successors to traditional conductors [17].

However, it was soon observed that when individual CNTs are formed into macroscopic ensembles such as films or fibers, their performance radically declines [18]. This unfavorable consequence is caused by many extrinsic and intrinsic factors that limit the conductivity of such networks [19]. One solution to this problem is to dope the material either with electron-withdrawing or electron-donating species to cause p- or n-doping, respectively. The former includes electron-poor agents such as inorganic acids [20,21] or halogens [22,23]. Regarding the latter class, electron-rich chemicals containing nitrogen atoms [24,25] or alkali metals [23,26] are often employed. However, many of the effective dopants are hazardous and/or their effect is not stable in time, so their potential for commercial implementation is limited. It was recently shown that water vapor can act as a potent doping agent for CNTs [27,28,29,30]. Results of multiple studies validated that the electrical conductivity of macroscopic ensembles such as films or fibers increases considerably when they are exposed to conditions of high humidity. Still, the literature lacks scalable procedures to exploit this phenomenon to the fullest extent.

In this contribution, composite films from single-walled CNTs (SWCNTs) and ethyl cellulose (EC) were doped with steam using an iron that saturated these materials with water. The adsorption of water on the SWCNTs constituting a free-standing film resulted in a notable improvement to the charge transport capabilities of the network. The initial value of electrical conductivity was quadrupled, eventually reaching the level of 905 ± 34 S/cm. Importantly, the doping was durable, as evidenced by the fact that after a month from the treatment, the value of electrical conductivity diminished by only 3%. The remarkable stability of doping was ascribed to the beneficial presence of a minute amount of EC in the composite. The hygroscopic nature of the EC keeps the introduced water molecules trapped within the network, where they can n-dope the SWCNTs.

## 2. Materials and Methods

### 2.1. Chemical Compounds and Materials

Large-diameter SWCNTs (Tuball™, OCSiAl, Luxemburg), ethyl cellulose (EC; ethoxyl content 48%, 22 cps Acros Organics, Geel, Belgium), acetone (Avantor, Gliwice, Poland), and toluene (Avantor, Gliwice, Poland) were obtained from the indicated vendors. All of the chemical compounds were of p.a. class. Demineralized water used in the study was produced by the Millipore Elix 10 demineralization system (Millipore SAS, Molsheim, France). Its electrical conductivity was monitored during purification to ensure appropriate quality.

### 2.2. Preparation of EC/SWCNT and SWCNT Films

**Figure 1:**
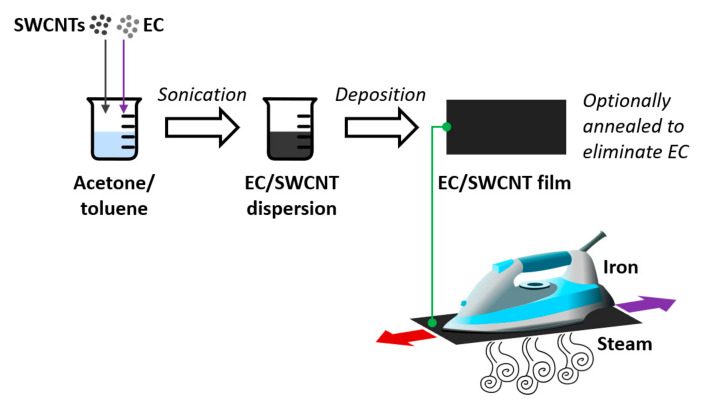
Preparation of ethyl cellulose (EC)/single-walled carbon nanotubes (SWCNT) and SWCNT free-standing films doped with water vapor.

The SWCNT films were prepared according to a method published by us [31] (Figure 1, top).

In brief, 490 mg of SWCNTs along with 10 mg EC, both dried in a desiccator, were introduced to ice-cold 80 mL of acetone/toluene mixture (1:1, w/w). The mixture was then homogenized by ultrasonication at a 100% amplitude until reaching uniform dispersion after 10 min of processing (UP200St sonicator, Hielscher, Teltow, Germany). The obtained EC/SWCNT dispersion was deposited onto a Kapton foil and left to dry in the ambient air. Low adhesion of the nanocarbon material to the substrate enabled straightforward delamination of the free-standing films from the surface. Where indicated, the films were briefly exposed to a high temperature to remove the EC. The thickness of the films was ca. 100 μm.

### 2.3. Doping of EC/SWCNT and SWCNT Films with Water Vapor

Doping of the films was carried out by ironing them with an iron (GC4532/20, Philips, Eindhoven, Netherlands). The temperature of the iron soleplate was measured to be 120 °C. In each case, a 9 cm × 9 cm SWCNT film sample (with and without EC) was ironed for 3 min at these settings. A quantity of 10 mL of water vapor was injected into the ensemble during this time (Figure 1, bottom). The ironing process reduced the thickness of the films by up to 10%. This effect was taken into account during determination of electrical conductivity.

### 2.4. Characterization

A Scanning Electron Microscope (SEM, FEI Quanta 250 FEG, Hillsboro, OR, USA) running at 5 kV acceleration voltage was used to study the microstructure of the films. Due to the highly-conductive nature of the EC/SWCNT samples, they were not sputtered with metal.

Raman spectroscopy (inVia Renishaw Raman microscope, λ = 633 nm, Wotton-under-Edge, UK) was employed to track possible structural and electronic changes to the material upon processing. The spectra were acquired from 10 to 3200 cm^−1^. Several accumulations were recorded for each examined sample in multiple areas. These measures were necessary to minimize the background noise and ensure that the collected data is statistically significant, respectively. Laser power was kept to a minimum (0.01% beam intensity) while the spot size was about 3 μm in diameter. Both of these measures were taken to make sure that illumination does not overheat the sample, which could alter the shape of the spectra [32]. Mean values of I_D_/I_G_ ratios were presented along with calculated standard deviations. Moreover, the G peak maximum positions were established for selected samples before and after exposure to water vapor.

A four-probe method was used to measure the electrical conductivity of the material. Specimens of 3 mm × 20 mm size, cut out from the films, were attached to custom-designed sample holders consisting of Cu tape terminals. Ag conductive paint was engaged to ensure appropriate mechanical and electrical contact. The samples were measured with a source meter (Keithley 2450 SourceMeter, Cleveland, OH, USA). Conductance was recalculated to conductivity by taking into account the sample area and thickness. The latter was determined with a micrometer screw gauge (Electronic Universal IP54, Linear Tools, Dunstable, UK).

## 3. Results

**Figure 2:**
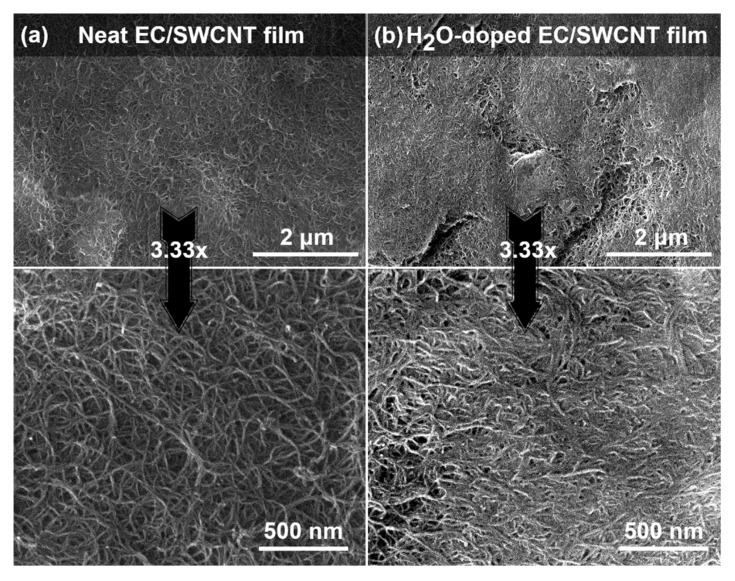
SEM micrographs of EC/SWCNT free-standing films (**a**) before and (**b**) doping with water vapor along with corresponding magnifications.

The study was initiated by an investigation of the microstructure of the EC/SWCNT films by SEM (Figure 2). The results showed that the starting material (Figure 2a) contained interweaved SWCNTs and their bundles, which were distributed therein without any degree of anisotropy. The material appeared porous, as expected for SWCNT networks [33]. Importantly, only negligible amounts of contamination such as amorphous carbon or metallic species could be discerned. Upon exposure to steam, considerable modification of the microstructure can be observed (Figure 2b). The packing of the material appeared higher as many of the voids were removed during the process. One of the major impediments for effective charge propagation through nanocarbon networks is the problem of contact resistance [19,34]. The empty space in the material is filled with air, which is insulating, thereby disturbing the electrical continuity. Consequently, better contact between SWCNTs should improve the electrical conductivity of the material for purely geometrical reasons.

Electrical properties of the material can also be enhanced by overcoming the intrinsic limitations to the charge transport in SWCNTs. The ability of SWCNTs to propagate charge is highly dependent on their crystallinity. Imperfections such as dislocations or functional groups make a strongly negative impact on the electronic transport properties of SWCNTs [35,36]. A convenient technique to quantify how pristine the SWCNTs are is Raman spectroscopy. Typically, the ratio of the intensities of the D-band (disorder-induced mode) and G-band (tangential mode of sp^2^ carbon atoms) is used to reveal such information [37,38].

**Figure 3:**
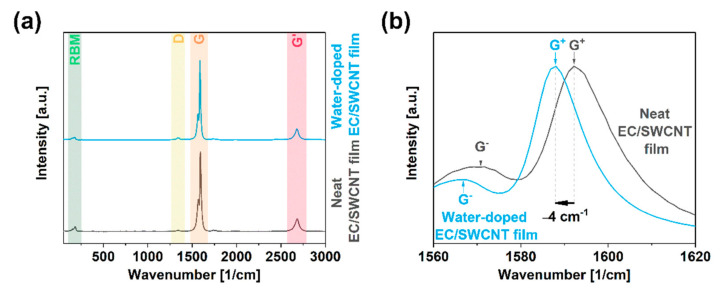
(**a**) Full Raman spectra of EC/SWCNT free-standing films before and after doping with water vapor, (**b**) close-up view of the G peak area.

The Raman analysis showed that the parent material used for the study was of very high quality (Figure 3a). The I_D_/I_G_ ratio was remarkably low and amounted to only 0.016 ± 0.004. The detected Radial Breathing Mode (RBM) in the enclosed spectra confirmed the single-walled character of the material. Exposure to water vapor at high temperature did not deteriorate the quality of the SWCNTs. The I_D_/I_G_ ratio remained at the same level of 0.018 ± 0.003. Considering the measurement uncertainties of these two values and the very low values of the I_D_/I_G_ ratio of both the samples, these slight differences were disregarded.

Furthermore, a detailed analysis of the G band line shape indicated electronic changes to the material upon incorporating water molecules into the material. The shape of the G mode was bimodal, so it was deconvoluted into G^−^ and G^+^ components. First of all, the presence of these two features confirmed the single-walled nature of the examined SWCNTs (Figure 3b) [39]. Secondly, the position of the G^+^ peak was used to study the doping character of the material [40]. The position of this feature in neat EC/SWCNT films was 1592 cm^−1^, while after the steam treatment, it shifted to 1588 cm^−1^. The shift in this direction is typically challenging as the dopant has to oppose the p-doping action of oxygen under the ambient conditions, naturally blue-shifting the spectrum [27]. Therefore, the presence of this phenomenon strongly suggested that the inherent conductivity of SWCNTs should be enhanced. Additionally, the direction of the shift induced by water indicated that the dopant had an electron-donating character, which stayed in accordance with previously published findings [28]. It has to be stressed that a shift to the G feature can also result from the modification of the microstructure when strain is introduced to nanocarbon networks [41]. However, we did not find this effect in force in this work. The position of the G+ peak barely moved when the films were ironed in the steam-free mode. Slight changes not exceeding 1 cm^−1^ were ascribed to the measurement uncertainty.

It is important to note that the impact of water on CNTs remains the subject of the scientific debate as its role seems to be dependent on the type of treated CNTs, their surface chemistry, and the degree of purity. For instance, Lekawa-Raus et al. showed that there is no convincing evidence to support either n-doping or p-doping when MWCNTs are exposed to water vapor [27].

**Figure 4:**
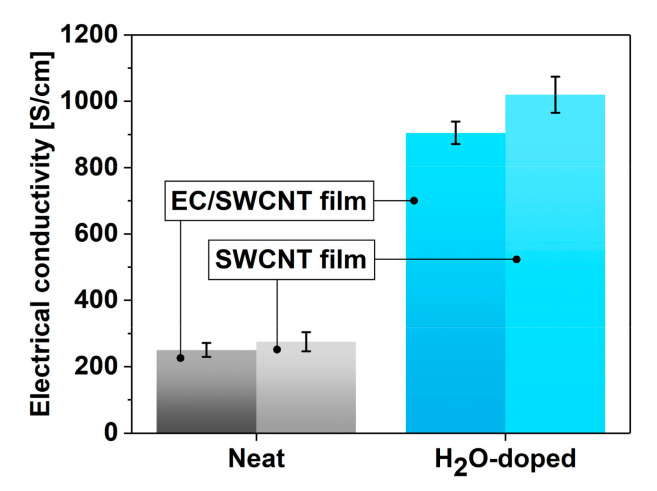
Comparison of the impact of water vapor addition on the electrical conductivity of EC/SWCNT and SWCNT films.

To confirm the utility of these findings, the electrical conductivity of the EC-SWCNT composites was measured before and after exposure to steam (Figure 4). EC-free SWCNT material was also studied as a reference. In the former case, the electrical conductivity of unprocessed nanocarbon composites was at the level of 250 ± 21 S/cm. After the doping process, the conductivity increased to 905 ± 34 S/cm, which is almost quadruple the starting value.

When the electrical conductivity of pure SWCNTs was measured, it amounted to 275 ± 29 S/cm. Therefore, the presence of EC did not make a negative impact on the charge propagation in undoped SWCNT networks, as these values stayed within the level of uncertainty. However, when the EC-free SWCNT ensemble was exposed to water, the initially measured electrical conductivity of the material was the highest among all the tested formulations, i.e., 1020 ± 54 S/cm. The record level of performance can be explained by unconstrained access to SWCNTs for water molecules. In this configuration, there are no physical barriers, such as EC of relatively large MW, which could block the access to the SWCNTs to dope them.

**Figure 5:**
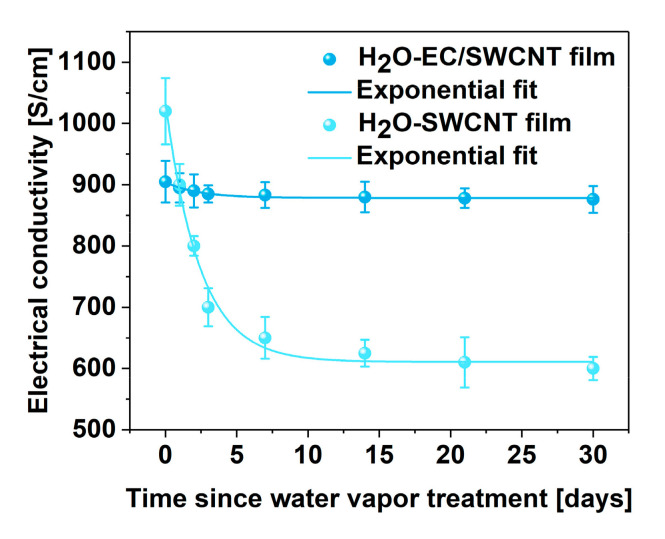
The change of electrical conductivity of EC/SWCNT and SWCNT films doped with water vapor over 30 days.

The critical attribute of a successful doping strategy is that its effects are time-invariant. Therefore, the durability of the proposed tactic was studied. The electrical conductivity of the SWCNT and EC/SWCNT films doped with water vapor were examined over the course of 30 days (Figure 5). In the case of the former material, although the initial electrical conductivity was the highest, it was quickly diminished. After a month, the electrical conductivity decreased from 1020 ± 54 S/cm down to 601 ± 19 S/cm in an exponential manner, which was a 41% reduction. Conversely, when the former material containing a minute fraction of EC was tested for a prolonged time, the electrical conductivity was decreased only slightly, i.e., from 905 ± 34 S/cm to 876 ± 22 S/cm. When recalculated into relative values, this gave only a 3% reduction in electrical conductivity.

**Figure 6:**
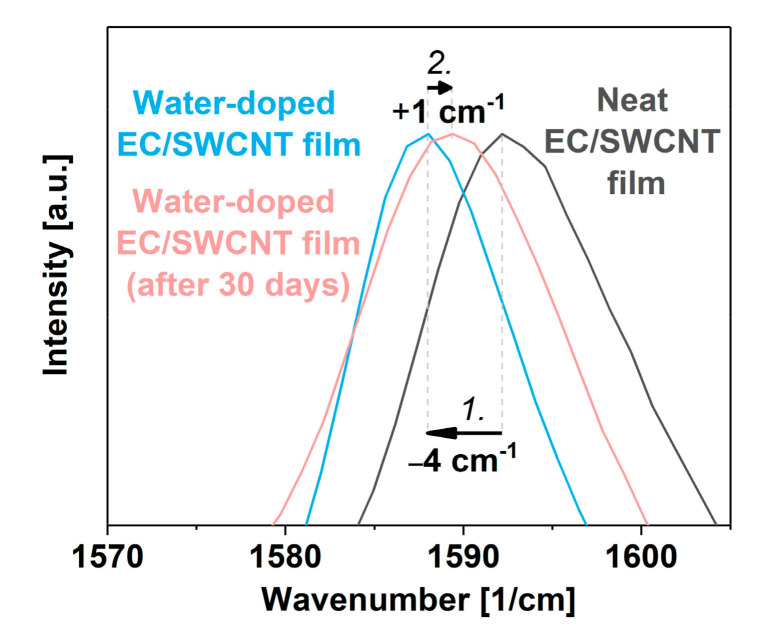
Analysis of the G^+^ peak area of the neat EC/SWCNT films, freshly doped with water vapor (1.) and after 30 days since the treatment (2.).

The change of electrical conductivity was accompanied by a slight shift in the position of the G^+^ peak in the Raman spectrum (Figure 6). The G^+^ peak maximum was shifted back in the direction of that of the undoped material ca. 1 cm^−1^, resulting from the loss of a small amount of the loosely bound dopant to the ambient.

We hypothesize that the tendency of the water dopant to desorb was dictated by the composition of the material. The small amount of EC in the composite (1:49 with respect to SWCNTs) determined whether the water molecules would be labile or not. In addition to its hydrophobic nature, which makes it interact well with CNTs, EC, and similar cellulose derivatives, they are slightly hygroscopic [42]. Therefore, incorporating even a minute amount of such a material in a composite appeared to preserve the water molecules in the SWCNT network. As a consequence, the electrical conductivity of the material was increased much more permanently.

## 4. Conclusions

To sum up, we showed how water vapor could be simply deposited inside of macroscopic ensembles based on SWCNTs. Thin free-standing films doped with water experienced a considerable increase in their electrical conductivity, which quadrupled after an undemanding post-synthesis processing. The largest improvement in conductivity was for neat SWCNT films, which upon steam addition reached 1020 ± 54 S/cm. However, for this formulation, the effect was not stable and faded in time. After 30 days, a 41% reduction in electrical resistance was witnessed. On the other hand, SWCNT films with a small amount of EC (1:49 with respect to SWCNTs) also exhibited a strong boost to the electrical conductivity, but the enhancement was permanent. The initially obtained conductivity of 905 ± 34 S/cm decreased by just 3% after a month since the treatment. The findings illustrate that adding a small amount of hygroscopic polymer such as EC to form a composite with nanocarbon is favorable in this situation.

For any post-synthetic strategy aimed to improve the properties of nanocarbon materials, primarily, they should be scalable and easily implementable. Equally essential factors to consider while developing modification routines for nanomaterials are sustainability and toxicity of the employed chemicals. The presented concept is straightforward and fulfills all these conditions. It involves a benign chemical compound (water), which can be effortlessly introduced to the material to obtain tangible benefits at minimal cost, effort, and environmental burden.
